# ATP Binding to p97/VCP D1 Domain Regulates Selective Recruitment of Adaptors to Its Proximal N-Domain

**DOI:** 10.1371/journal.pone.0050490

**Published:** 2012-12-03

**Authors:** Wei Sheng Chia, Diana Xueqi Chia, Feng Rao, Shoshana Bar Nun, Susana Geifman Shochat

**Affiliations:** 1 School of Biological Sciences, Nanyang Technological University, Singapore, Singapore; 2 Department of Biochemistry and Molecular Biology, George S. Wise Faculty of Life Sciences, Tel Aviv University, Tel Aviv, Israel; Helmholtz Centre for Infection Research, Germany

## Abstract

p97/Valosin-containing protein (VCP) is a member of the AAA-ATPase family involved in many cellular processes including cell division, intracellular trafficking and extraction of misfolded proteins in endoplasmic reticulum-associated degradation (ERAD). It is a homohexamer with each subunit containing two tandem D1 and D2 ATPase domains and N- and C-terminal regions that function as adaptor protein binding domains. p97/VCP is directed to its many different functional pathways by associating with various adaptor proteins. The regulation of the recruitment of the adaptor proteins remains unclear. Two adaptor proteins, Ufd1/Npl4 and p47, which bind exclusively to the p97/VCP N-domain and direct p97/VCP to either ERAD-related processes or homotypic fusion of Golgi fragments, were studied here. Surface plasmon resonance biosensor-based assays allowed the study of binding kinetics in real time. In competition experiments, it was observed that in the presence of ATP, Ufd1/Npl4 was able to compete more effectively with p47 for binding to p97/VCP. By using non-hydrolysable ATP analogues and the hexameric truncated p97/N-D1 fragment, it was shown that binding rather than hydrolysis of ATP to the proximal D1 domain strengthened the Ufd1/Npl4 association with the N-domain, thus regulating the recruitment of either Ufd1/Npl4 or p47. This novel role of ATP and an assigned function to the D1 AAA-ATPase domain link the multiple functions of p97/VCP to the metabolic status of the cell.

## Introduction

p97, also known as valosin-containing protein (VCP), is a member of the AAA (ATPase associated with various cellular activities) ATPase family. Structurally, as most AAA-ATPases, p97/VCP adopts a toroidal hexameric structure comprising six identical 90 kDa subunits arranged in a ring with a diameter of 160 Å, height of 80 Å and a central pore [1]–[6]. Each monomeric subunit consists of a major adaptor protein-recruiting N-terminal domain (N-domain), two tandem AAA-ATPase domains, D1 and D2, each with Walker A and Walker B motifs responsible for ATP binding and hydrolysis, respectively, and a minor adaptor protein-recruiting C-terminal domain, which contributes to hexamer stability [7]. In the homohexamer, the D1 domain of each monomer is stacked on top of the D2 in a head to tail packing, resulting in a D1 “disc” on top of a D2 “disc” having two faces with six ATP binding sites each. The p97/VCP D1 ATPase domain has low hydrolytic activity, while ATP binding to D1 was reported to play a role in accelerating the p97/VCP homo-hexamerization [8]. The bulk of the ATPase activity, which is believed to generate most of the force that is required for p97/VCP functions, is provided by the D2 domain [9]–[11]. Structural data suggest that chemical energy derived from ATP hydrolysis is converted into mechanical work by transmission of conformational changes generated by D2, through the D1–D2 linker, to displace the N-domain [4], [6], [10], [12]–[17].

p97/VCP is an essential and abundant protein that is ubiquitously expressed and is conserved throughout evolution from archaea to mammals [18] In a living cell, p97/VCP resides in both the nucleus and the cytoplasm, and has been estimated to constitute up to 1% of the cell’s total protein content. Being an AAA-ATPase, p97/VCP is implicated in multiple cellular processes, including molecular segregation, gene regulation, cell cycle regulation and spindle disassembly, homotypic membrane fusion, intracellular trafficking and protein quality control via ubiquitin-proteasome-mediated degradation [18]–[22] Of the multiple functions of p97/VCP, its involvement in the endoplasmic reticulum-associated protein degradation (ERAD) pathway is the best characterized [23]–[25]. In ERAD, p97/VCP is proposed to be the main molecular ratchet for the energy-dependent extraction of misfolded proteins out of the ER [9], [25]–[27]. The key role of p97/VCP in protein homeostasis is indicated by its association with various diseases including cancer and with protein aggregates characteristic of proteinopathies linked to many neurodegenerative diseases [28]–[33]. A rare multisystem degenerative disorder, known as inclusion body myopathy Paget’s disease of the bone and frontotemporal dementia (IBMPFD), is caused by dominantly inherited missense mutations in the *VCP* gene encoding p97/VCP [34], [35]. Mutations in p97/VCP are also implicated in familial amyotropic lateral sclerosis (ALS) [36].

The mechanism by which p97/VCP is engaged in multiple tasks is poorly understood. Its pleiotropic performance is likely the outcome of a basic segregase activity that extracts proteins from protein complexes and cell membranes, a function that is needed in many different cellular processes [37], [38]. It is also established that specificity is achieved by a plethora of adaptor proteins that contain several identified p97-interacting motifs. By associating with p97/VCP, mostly through its N-domain, these adaptor proteins engage the AAA-ATPase in particular functions [39]. However, despite several structural studies, it remains unclear how the recruitment of the increasing number of various adaptor proteins is regulated [40]. This is exemplified by the two best characterized adaptor proteins, p47 and Ufd1/Npl4, with the latter being a heterodimer of ubiquitin-fusion degradation protein 1 (Ufd1) and nuclear localization protein 4 (Npl4), and commonly referred to as UN. These two types of adaptor proteins bind to the N-domain of p97/VCP in a mutually exclusive manner, directing p97/VCP to two different processes. Association with p47 links p97/VCP to its substrate syntaxin 5 in post-mitotic homotypic Golgi fragments fusion, whereas UN binding directs p97/VCP to ERAD, ubiquitin and nuclear transport [41]–[43].

To better understand how the interactions of the various adaptor proteins with p97/VCP are regulated, we performed a series of binding assays, utilizing surface plasmon resonance (SPR) biosensor technology. SPR biosensors offer the advantage of real time monitoring of binding and dissociation events, which can be followed under different conditions. We focused on the effects of nucleotides, at physiological concentrations, on the binding of p47 and UN to the N-domain and the contribution of the proximal D1 domain to regulating the interactions of these adaptor proteins with p97/VCP. Based on the results of our competition assay, we conclude that in the presence of ATP, UN competes more effectively with p47 for p97/VCP binding. With the use of the p97-N-D1 fragment, we were able to show that binding rather than hydrolysis of ATP to the D1 domain is sufficient for regulating this competition. Together with the effect of ATP that enhanced binding to p97/VCP of UN but not of p47, we propose that ATP binding to the D1 domain plays a role in regulating the exclusive recruitment of either UN or p47 to the p97/VCP proximal N-domain and thus in directing p97/VCP to either ERAD or homotypic fusion, respectively. Our results assign a novel role to the D1 AAA-ATPase domain and link p97/VCP function to the ATP level and the metabolic status of the cell.

## Results

### Protein Purification

**Figure 1:**
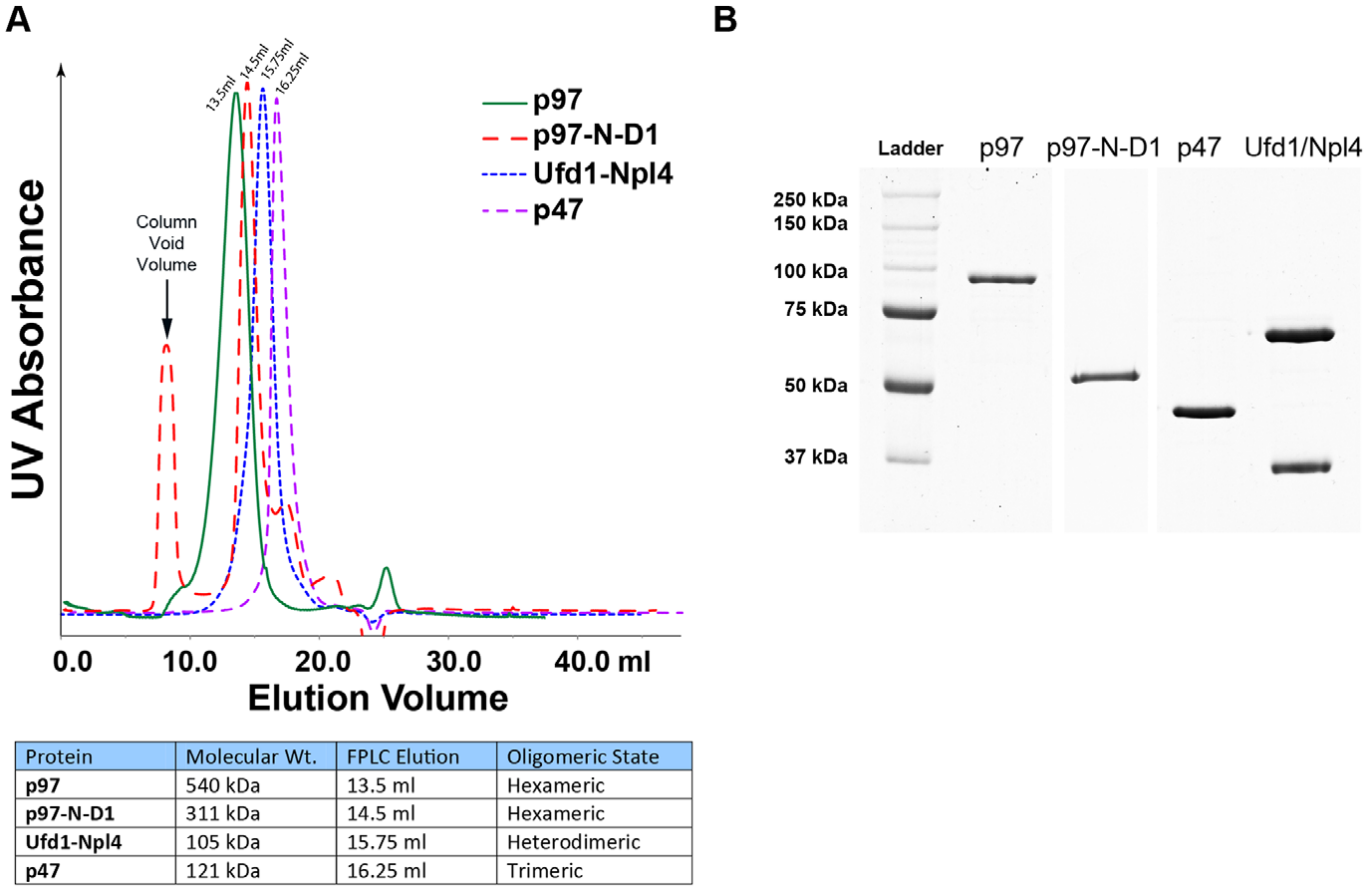
Purification of p97/VCP and adaptor proteins. His-tagged full length p97 or p97-N-D1 fragment or adaptor proteins Ufd1/Npl4 (UN) or p47 were expressed in *E. coli Rosetta DE3* (Novagen) and purified under native conditions using nickel affinity chromatography and gel filtration. UN was co-purified as a heterodimer through the His-tag of Ufd1. (A) UV peak fractions at the appropriate elution volumes on Superose 6, corresponding to the respective oligomeric states of each protein that were used for Biacore binding assays (hexameric 13.5 ml fraction for full length p97/VCP and 14.5 ml fraction for p97-N-D1) (B) The purified proteins were resolved by SDS-PAGE and stained with Coomassie brilliant blue. For calibration of the Superose 6 column, see Figure S1.

The recombinant p97/VCP, p97-N-D1, p47 and Ufd1/Npl4 used in our SPR, DSF and DLS assays were expressed in bacteria and purified to homogeneity (Figure 1), as described in Experimental Procedures. The calibration of the Superose 6 column used for purification is shown in Figure S1 and a table presenting the elution volumes and the oligomeric states of the purified proteins are shown in Figure 1. Ufd1/Npl4 heterodimer was eluted in gel filtration at an apparent molecular mass of 200 kDa due to its shape, despite being only 105 kDa in molecular weight, similar to the observations of Bruderer *et al.* [44]. p47 was eluted as a homotrimer at 16.25 ml, identical to the purifications reported by Kondo *et al*. [42]. p97/VCP was eluted as a very stable homohexamer and p97-N-D1 fragment was eluted as two different fractions, a minor monomeric fraction at 17.7 ml and a homohexameric fraction at 14.5 ml. The homohexameric fraction was used for all our assays. To verify that the proteins we used were hexameric, dynamic light scattering (DLS) was performed on both full length p97/VCP and p97-N-D1 fragment as well.

### The Affinity of the Interactions of p97/VCP with Either p47 or UN

**Figure 2:**
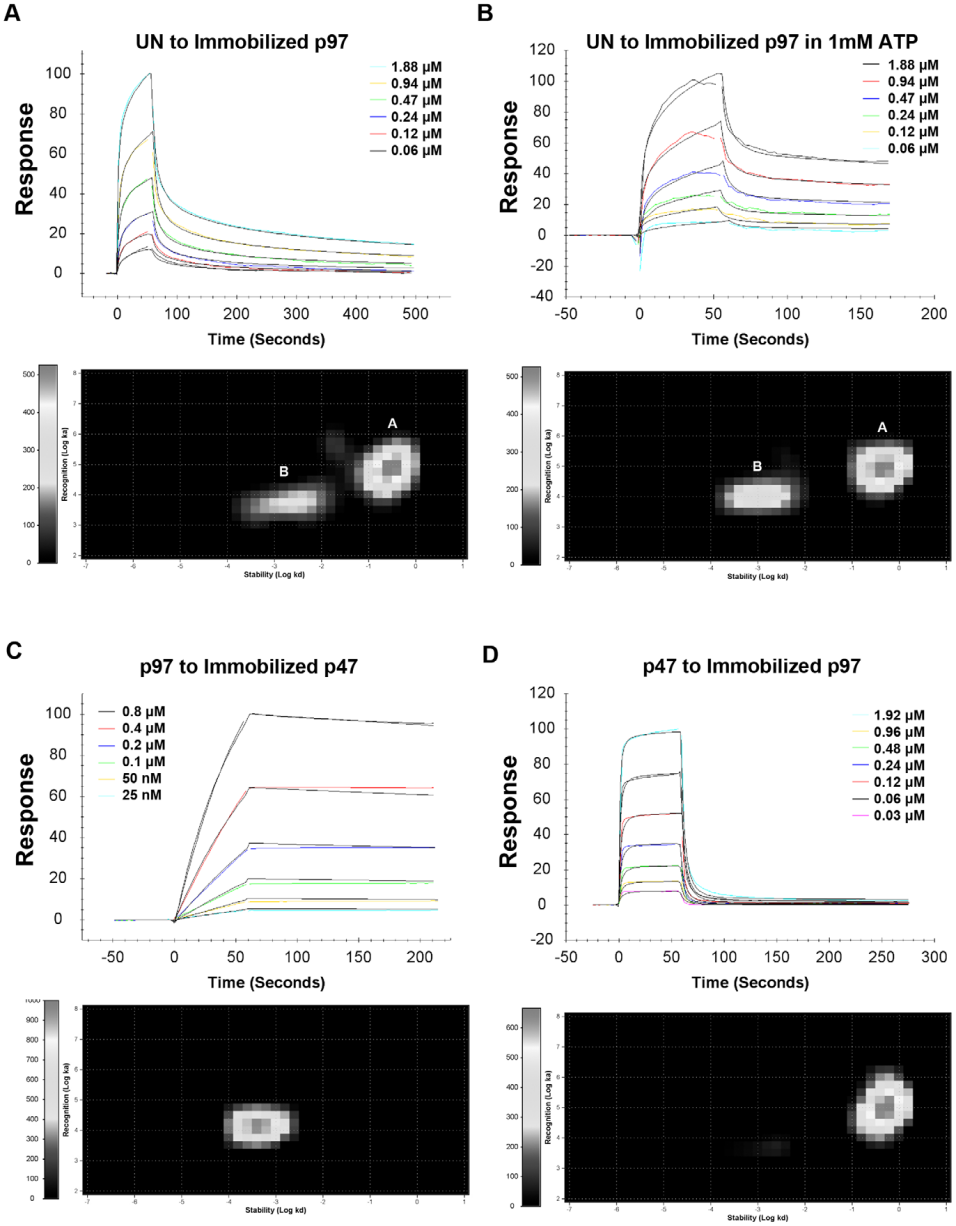
Binding affinities of the interactions between p97/VCP and its adaptor proteins. p97/VCP was immobilized on a CM5 sensorchip surface, using amine coupling procedure, and a concentration series of p47 (two fold dilutions from 1.92 μM) or UN (two fold dilutions from 1.88 μM) were injected over the immobilized surface at a flow rate of 30 μl/min, at 25°C, using PBS as the sample and running buffer. Sensorgrams and the corresponding interaction maps for the p97/VCP-UN interaction without ATP (A), with ATP during UN association and dissociation (B) and for p97/VCP-p47 interactions when p47 is immobilized (C) and when p97/VCP is immobilized (D) are shown. Interaction Map Analysis shows that the interaction between UN and p97/VCP is complex, with a major component with ∼5 μM affinity, and a second component with ∼400 nM affinity. When ATP is added, the affinity of the second component increases to 100 nM. For the interaction between p97/VCP and p47, only one kinetic component was found, and the interaction could be fitted to a Langmuir 1∶1 interaction model, with ∼31.3 nM affinity when p47 is immobilized and p97 injected across (C) or ∼5 μM affinity when p97 is immobilized and p47 injected across (D).

Using a Biacore 3000 instrument, an attempt was made to determine the affinity of the interactions of p97/VCP with either p47 or UN. For that purpose, p97/VCP was immobilized on the CM5 sensorchip surface and concentration series of p47 or UN were injected over the immobilized surface at two-fold dilutions, as shown in Figure 2. For the interaction between p97/VCP and UN shown in Figure 2A, the sensorgrams could not be fitted with a Langmuir 1∶1 model, showing a multiphasic behavior. It implies that the interaction is complex even at a low immobilization of p97/VCP, which did not show mass transfer limitation (Figure S2).

To determine the components of the p97/VCP-UN interaction, we have submitted the data of the binding curves for the interaction between UN and p97/VCP to an Interaction Map Analysis service, where advanced mathematical fittings are performed computationally on the curves. From the results obtained from this analysis, illustrated in Figure 2A, it is evident that the interaction is complex and can be represented by two independent processes, a dominant weak-affinity component (K_D_ of approximately 5 μM), represented by peak A, which is the major interaction of p97/VCP and UN, and an additional smaller but stronger interaction component of approximately 400 nM affinity (peak B). Given the complex nature of this interaction, it is impossible to provide one single affinity value but merely to display the Interaction Map Analysis.

For the p97/VCP-p47 interaction, the sensorgrams obtained from the binding of p47 to immobilized p97/VCP or *vice versa* were fitted with a Langmuir model and using Interaction Map Analysis they showed a single kinetic component (Figure 2C, D). However there are kinetic and affinity differences depending on the immobilized interactant. When p47 was immobilized and p97/VCP passed across, a strong interaction was observed (31.3 nM). A strong interaction between p97/VCP and p47 had been reported by Kondo *et al.* [42], who showed that p47 could not be removed from p97/VCP in their gels, and Dreveny *et al.* [3] stated that the interaction between p97/VCP and p47 was stable up to 0.5 M KCl. This could be due to one p97/VCP homohexamer interacting with more than one immobilized p47, having a higher interaction stoichiometry contributing to avidity. When p97/VCP was immobilized and p47 was passed across as an analyte, the kinetics of the interaction presented fast association and dissociation, with a K_D_ of around 5 μM. In this case, one could envision p47 interacting with individual sites on p97/VCP and falling off, instead of locking in as a stable complex.

Given the complexity of the interactions between p97/VCP and UN, which renders quantitative measurements and comparisons difficult, we have adopted a qualitative approach. In our attempts to study adaptor preference, we perform competition assays between p47 and UN in their binding to p97/VCP.

### UN Competes More Effectively with p47 for Binding to p97/VCP in the Presence of ATP

**Figure 3:**
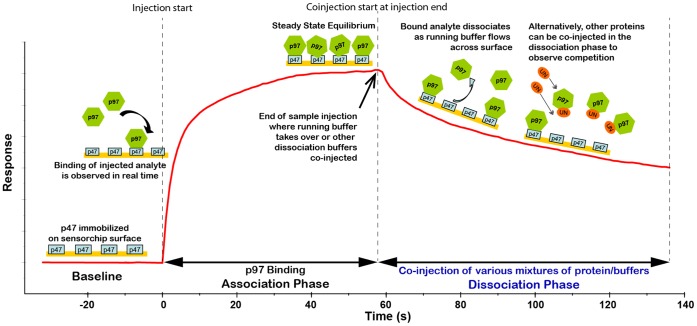
The design of the competition experiments using the Biacore 3000 instrument. The cartoon depicts the different steps in each binding cycle designed to observe competition between Ufd1/Npl4 (UN) and p47 for binding to p97/VCP in real time. The first binding partner, p47, was immobilized on a CM5 sensorchip by amine-coupling procedure to a level of 2000 RU. Injection of p97/VCP across the p47 surface allowed the capture of p97/VCP to a level of 500 RU before UN was coinjected and p97/VCP dissociation was monitored. At the end of each experiment, the surface was regenerated by a 3 sec pulse of 50 mM NaOH. The capture of p97/VCP could then be repeated with UN coinjected at different concentrations or in the presence of ATP.

To gain insights into the regulation of recruitment of the various adaptor proteins of p97/VCP, an SPR-based competition assay was designed and carried out using a Biacore 3000 Instrument (Figure 3). In this assay, we were able to investigate the competition between p47 and UN for binding to p97/VCP, using a customized protocol (Figure S3) that allowed high reproducibility and accuracy in real time observations.

**Figure 4:**
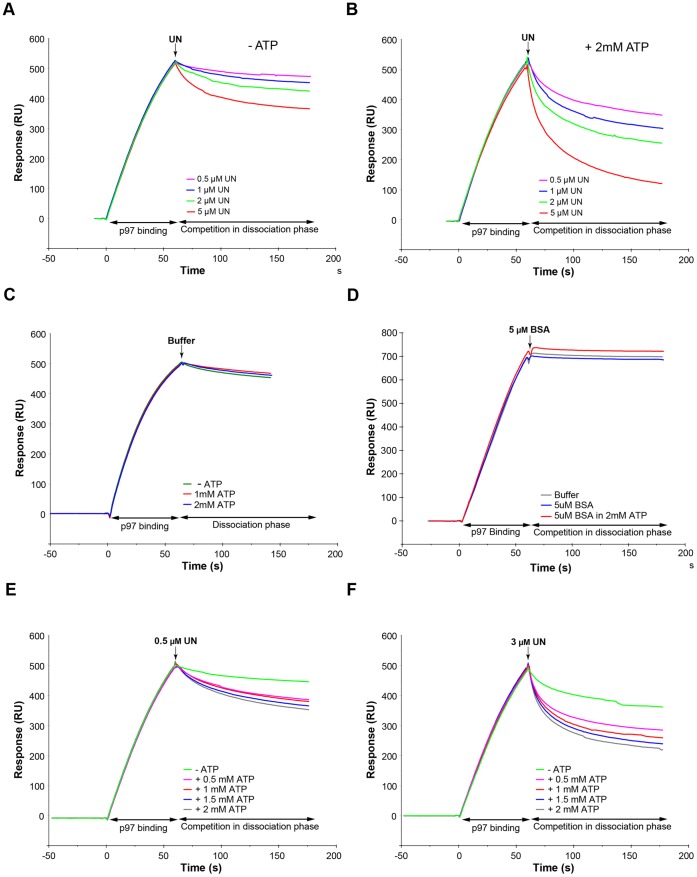
Ufd1/Npl4 competes with p47 for binding to p97/VCP more effectively in the presence of ATP. p47 (2000 RU) was amine-coupled onto a flowcell of a CM5 sensorchip and p97/VCP (500 RU) was repeatedly captured by the immobilized p47 as depicted in Figure 3 and described in Experimental Procedures. UN at the indicated concentrations was coinjected across the surface, in the absence (A) or presence (B) of 2 mM ATP. In control experiments, 2 mM ATP with no UN (C) or 5 µM BSA instead of UN (D) were coinjected. UN was coinjected at fixed concentrations of either 0.5 µM (E) or 3 µM (F) in the presence of the indicated ATP concentrations.

To follow the competition between UN and p47 for p97/VCP, we selected p47 for immobilization on the sensorchip surface. This was based on the high stability of p47 and its resistance to surface regeneration conditions, which allowed tens of analyte injections and regeneration cycles with minimal surface deterioration. Approximately 500 response units (RU) of p97/VCP were initially captured on the p47-immobilized surface (Figure 4). During the dissociation phase, various concentrations of UN were injected in the absence (Figure 4A) or presence (Figure 4B) of ATP. Clearly, in the presence of ATP, UN competed with p47 for p97/VCP binding more effectively. When 2 mM ATP was co-injected, 0.5 μM of UN was as effective as 5 μM UN in the absence of the nucleotide (compare Figure 4A and B). It indicates a ten-fold increase in the capability of UN to compete with p47. Note that without UN, ATP had no effect on the dissociation of p97/VCP from p47 (Figure 4C). The specificity of this competition was confirmed by substitution of UN with bovine serum albumin (BSA), where BSA failed to remove p97/VCP from the p47 surface (Figure 4D). Finally, by varying both UN and ATP concentrations, we demonstrated that ATP influenced the competitive binding in a concentration-dependent manner, as the enhanced competition by elevated amounts of UN was further improved in correlation with increasing ATP concentration (Figure 4E, F).

### ATP Binding, not Hydrolysis, to the D1 Domain of p97/VCP Regulates Adaptors' Competition

**Figure 5:**
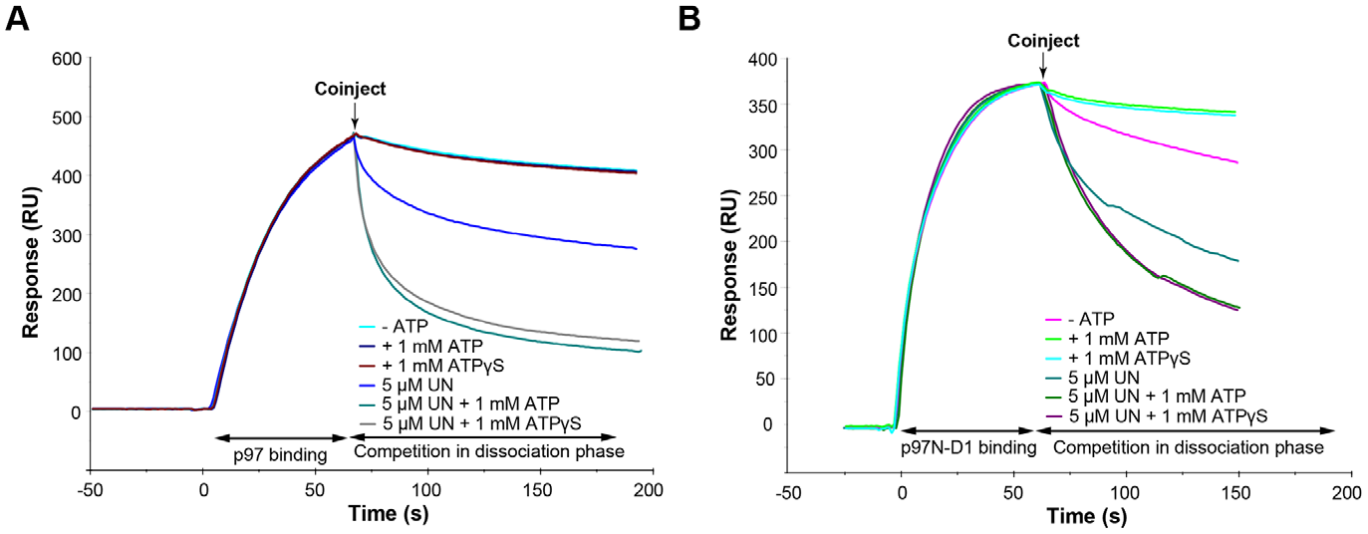
ATP binding to the p97/VCP D1 domain regulates the adaptor protein competition. Full length p97/VCP (A) or p97-N-D1 fragment (B) were captured on the p47 surface to a level of 500 RU. 5 μM of UN was coinjected across the surface in the presence of 1 mM of either ATP or ATPγS, using the same experimental setup described in Figure 4,

To discern whether ATP binding or its hydrolysis is responsible for regulating the observed competition between the adaptor proteins, we utilized the non-hydrolysable ATP analogue ATPγS. Clearly, competition was as effective with the non-hydrolysable analogue as with ATP (Figure 5A). It indicates that ATP binding rather than its hydrolysis is responsible for the regulatory effect on the adaptors' recruitment.

Among the two ATPase domains present in p97/VCP, the D2 domain is implicated in most of the activities of p97/VCP that depend on ATP hydrolysis. We focused on the D1 domain, whose hydrolytic activity is much weaker, but it is in close proximity to the N-domain, the main p97/VCP adaptor recruiting region. Encouraged by recent publications on N-domain conformational changes upon ATP binding to p97-D1 [45], we hypothesized that the D1 domain is key to this regulation of the adaptor proteins competition. To rule out any contribution of the D2 domain, we carried out experiments with the p97-N-D1 fragment lacking the D2 domain. We found that ATP binding to the D1 domain itself was sufficient for regulating the adaptors’ interactions with p97/VCP. Clearly, the p97-N-D1 fragment (Figure 5B) faithfully mimicked the behavior of the full length p97/VCP (Figure 5A) vis-à-vis the enhancing effects of ATP or ATPγS on the ability of UN to compete with p47 for association with the N-domain of either the full length p97/VCP or its p97-N-D1 fragment.

### ATP Enhances the Binding of UN to p97/VCP but does not Affect the Binding of p47

**Figure 6:**
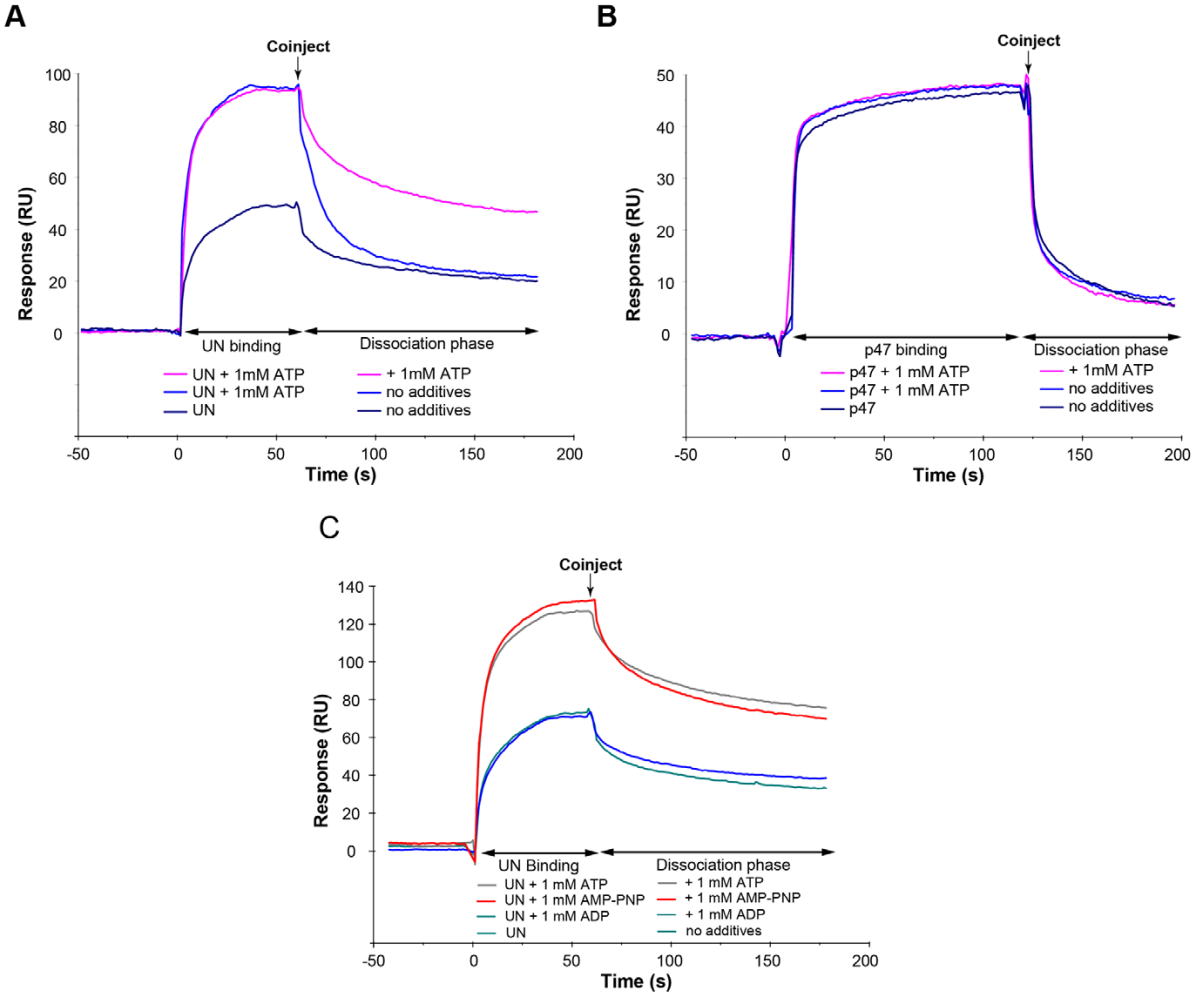
ATP binding to p97/VCP affects Ufd1/Npl4 association with p97/VCP. p97/VCP (1000 RU) was immobilized on a CM5 sensorchip and either UN (A)or p47 (B) were injected over the p97/VCP surface at a concentration of 0.15 μM in the absence or presence of 1 mM ATP during the binding and/or dissociation phases, as indicated. (C) 0.16 μM UN was injected across 1000 RU of immobilized p97/VCP in the absence or presence of 1 mM of ATP, AMP-PNP or ADP.

The results obtained from the competition experiments suggest that either ATP strengthens the binding of UN and/or weakens the binding of p47 to p97/VCP. To discern between these two mechanistic options, we tested the effects of ATP on the binding of either p47 or UN to p97/VCP. Our results showed unequivocally that ATP enhanced the binding of UN to p97/VCP (Figure 6A), while no effect of ATP could be detected on the binding of p47 (Figure 6B). Interaction Map Analysis of the sensorgrams of the p97/VCP-UN interaction showed that the second component of this interaction was strengthened from ∼400 nM in the absence of ATP to ∼100 nM affinity in the presence of 1 mM ATP (Figure 2B).

This finding provides a mechanistic explanation for the results obtained in the experiments showing the effect of ATP on the competition between UN and p47 for the binding to p97/VCP shown above. Again, when comparing the effects of ATP, ADP, and the poorly hydrolysable ATP analogue AMP-PNP, we noticed that both ATP and AMP-PNP exerted very similar effects, as both nucleotides strengthened the binding of UN to p97/VCP irrespective of hydrolysis (Figure 6C). It confirms that ATP binding rather than its hydrolysis is responsible for the improved binding of UN that, in turn, enhances its ability to compete more effectively with p47 for binding to p97/VCP.

### ATP Binding Exerts Conformational Changes on Homohexamers of p97/VCP or its p97-N-D1 Fragment

**Figure 7:**
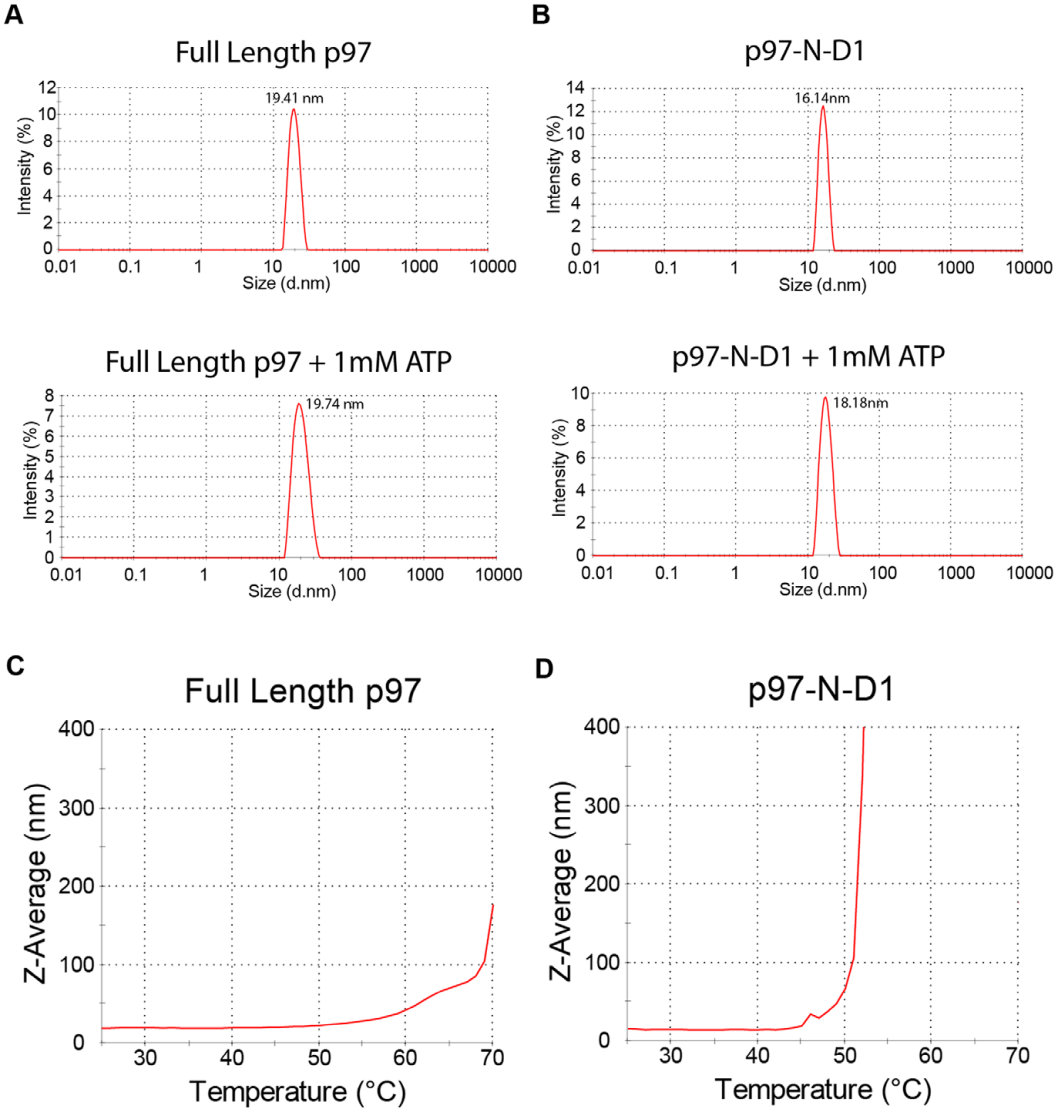
Dynamic light scattering demonstates the stability of p97/VCP and p97-N-D1 hexameres. Dynamic light scattering was performed on purified hexamers of either full length p97/VCP (A) or p97-N-D1 fragment (B) in the absence or presence of ATP. Upon addition of 1 mM ATP the hydrodynamic diameter of full length p97/VCP shifted from 19.41 nm to 19.74 nm and that of p97-N-D1 fragment shifted from 16.14 nm to 18.18 nm. The observed single peak homogeneities reflect no aggregation and stable hexamers. Both full length p97/VCP (C) and p97-N-D1 fragment (D) were subjected to thermal denaturation monitored by dynamic light scattering, showing no signs of hexamer dissociation when heated from 25°C to 70°C.

The effects of ATP on strengthening the binding of UN to p97/VCP and potentiating its ability to compete with p47 may reflect conformational changes in p97/VCP. To probe for such changes, we utilized differential scanning fluorimetry (DSF) that correlates fluorescence with protein unfolding at elevated temperatures. Prior to carrying out DSF experiments, both p97/VCP and p97-N-D1 were assessed by DLS experiments, which confirmed their homohexameric structures (Figure 7). Hydrodynamic diameters of 19.4 nm for the full length p97/VCP (Figure 7A) and of 16.14 nm for the p97-N-D1 (Figure 7B) indicate that the proteins we worked with were homohexamers. These parameters are within the range of the molecular diameters estimated from published crystal structures of homohexameric p97/VCP [4], [46], [47]. In the presence of ATP, there was a slight shift in diameter (Figure 7A, B), suggesting an extended/expanded conformation. Both p97/VCP (Figure 7C) and p97-N-D1 (Figure 7D) were subjected to thermal denaturation with their diameters being monitored by DLS. During the process, the homohexameric structures were stable without falling into their monomers, suggesting high thermodynamic stability of the homohexamers. To further ensure that we were observing the homohexameric structures, we attempted to denature p97/VCP with urea. Disassembly of the hexamers was induced by urea in a concentration-dependent manner, resulting in a hydrodynamic diameter of below 1 nm for the p97/VCP monomer (Figure S4).

**Figure 8:**
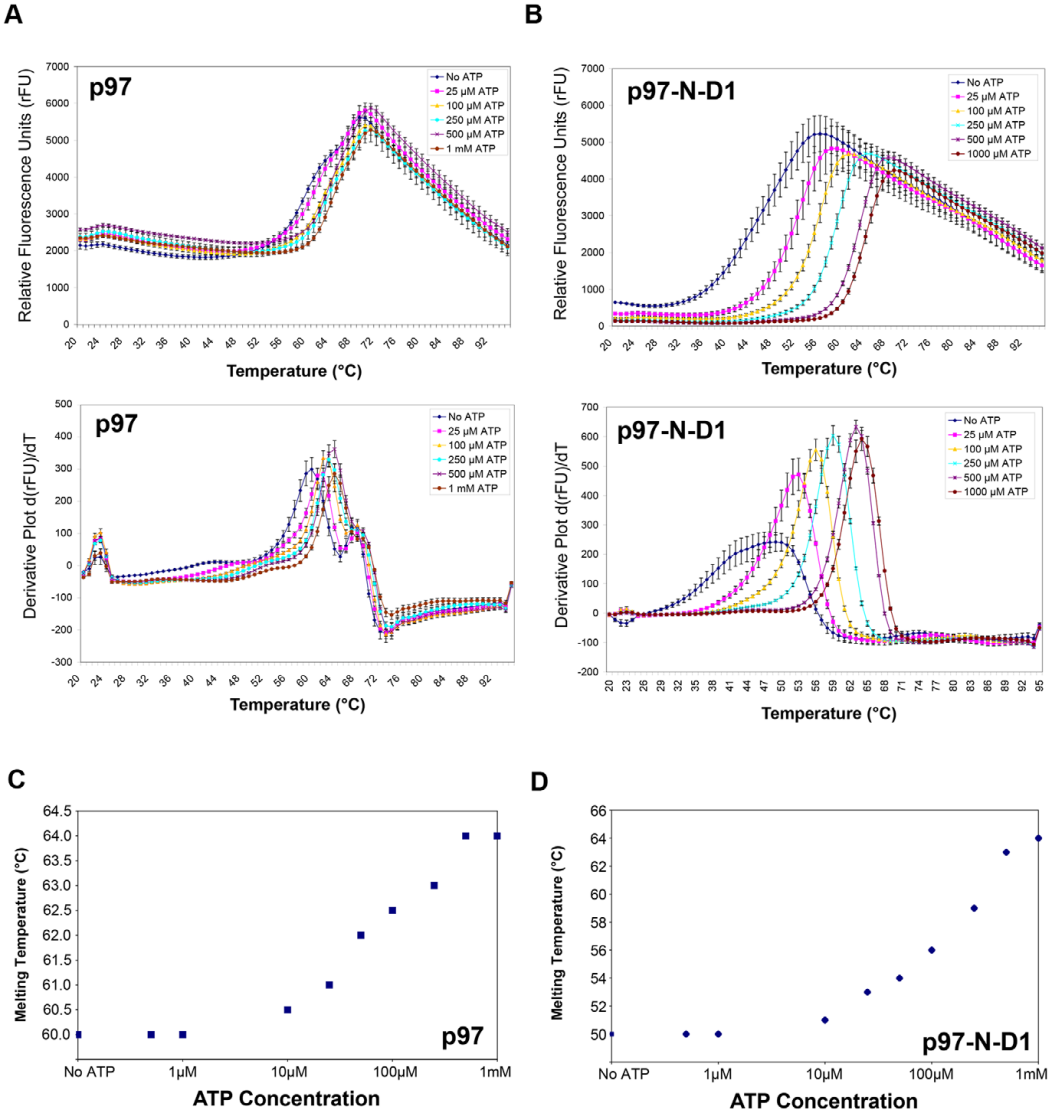
Conformational changes upon ATP binding to p97/VCP and p97-N-D1 observed by differential scanning fluorimetry. Hexamers of either full length p97/VCP (A) or p97-N-D1 fragment (B) were subjected to differential scanning fluorimetry in the absence or presence of 1 mM ATP. In the absence of ATP, the melting transition of full length p97/VCP hexamers was a two step process with the major component occurring at 60°C. Minor shifts were detected in the presence of ATP (A). p97-N-D1 hexamers had a melting temperature of 50°C in the absence of ATP, which was significantly shifted by 14°C to 64°C in the presence of ATP (B). Concentration-dependent thermostabilizations were observed by measurements of melting temperatures of full length p97/VCP (C) or p97-N-D1 fragment (D) at the indicated concentrations of ATP.

More importantly, the DSF results revealed that while for the full length p97/VCP there was a minor but reproducible change in the temperature at which the protein unfolded in the presence of 1 mM ATP, (Figure 8A), the p97-N-D1 fragment showed a more dramatic thermostabilization upon ATP binding, with the melting temperature shifting by 14°C, from 50°C in the absence of ATP, to 64°C in the presence of 1 mM ATP (Figure 8B). By titrating hexamers of both full length p97/VCP (Figure 8C) and the p97-N-D1 fragment (Figure 8D) with a series of ATP concentrations, we observed concentration-dependent stabilization. It suggests that ATP binding results in conformational changes that bring about stability against thermal unfolding.

## Discussion

p*97/VCP functions in a myriad of cellular processes to which this AAA-ATPase is targeted by the recruitment of various adaptor proteins. Most of these adaptors* bind to the same N-domain, either in a mutually exclusive way, as previously reported and shown here, or in hierarchical manner. Thus, the binding of the various adaptor proteins should be finely regulated. Several studies have provided the structural basis for the interaction between p97/VCP and its adaptor proteins [48]. p97-UN has been postulated to be a core complex platform for subsequent recruitment of other proteins into a functional mega protein complex, while p97-p47 forms another distinct core complex [49]. The competition between UN and p47 addressed in this study becomes important as it determines the population of core complexes and the consequent directing of p97/VCP to its various functions.

Our qualitative competition and binding assays show, for the first time, that the concentration of ATP and its binding to the D1 domain affect the affinity of the interactions between p97/VCP and either p47 or UN. Indeed, our attempts to determine the actual affinity values of p97/VCP to UN yielded multi-phasic curves that could not be fitted to a standard Langmuir interaction model. These multi-phasic binding SPR curves may be explained by a very recent paper by Bebeacua et al., demonstrating through cryo-electron microscopy that p97/VCP harbors several distinct binding sites for UN, with UN interacting via single or double binding site models [50]. Also, applying Interaction Map Analysis we were able to deconvolute the interaction between UN and p97/VCP into two main components, which make the comparison of the respective affinities to that of p97-p47 difficult. Irrespective of this shortcoming, we demonstrate that ATP exerts differential effects by strengthening the binding of UN, while not affecting the binding of p47, and therefore significantly enhancing the capacity of UN to compete with p47 for p97/VCP binding. The experiments performed with varying concentrations of ATP and UN, showing their co-dependence in competition with p47, suggest that in addition to the relative levels of these adaptor proteins, the cellular metabolic state referred to as ATP level affects the exclusive recruitment of either UN or p47 and the consequent function of p97/VCP in either ERAD or homotypic fusion. Moreover, with the use of the non-hydrolysable ATP analogues we attribute the effect of ATP to its binding rather than its hydrolysis.

While most ATPases have a single ATPase domain, it is not clear why each p97/VCP subunit contains two ATPases, the N-terminal D1 and the C-terminal D2. Especially intriguing is the role of the D1 domain, which has a low ATPase activity and is often ADP-bound, while the dominant ATPase activity is attributed to D2 that is observed in several nucleotide states [10]. ATP binding to D1, and not ATP hydrolysis, is implicated in accelerating hexamerization. Systematic analyses of mutations in p97/VCP revealed that the loss of ATPase activity of D2 led to the loss of function of the protein in vivo, while ATPase activity of D1 per se was not essential. Nevertheless, a mutation locking D1 in an ATP-bound form was exceptionally lethal, probably because this form of D1 changed an inter-domain interaction [51]. Here we attribute a novel role to the D1 domain as an ATP-sensing domain that regulates the binding of p97/VCP to its different adaptor proteins. To directly demonstrate the exclusive contribution of the D1 domain to adaptor recruitment, we have used the p97-N-D1 fragment, which lacks the major ATPase domain D2. Clearly, the D1 domain is responsible for regulating the ATP-enhanced competition between UN and p47 for the proximal N-domain of p97/VCP. Our results indicate that the D1-ATP-bound conformational state of the p97/VCP N-domain favors binding to UN over p47. Indeed, crystal structure and solution X-ray scattering studies by Tang et al. [45] confirm conformational changes in the N-domain upon binding of ATPγS.

Our results show that 0.5 µM of UN in the presence of 2 mM ATP competes with p47 for binding to p97/VCP as effectively as 5 µM of UN in the absence of ATP. This may be of high relevance in the cellular context, where ATP fluxes and gradients can exist locally or transiently [52]–[55]. Namely, under ATP-limiting conditions, p97/VCP favors binding to p47 over UN, whereas under ATP-abundance, p97/VCP binds preferably to UN. Recalling that p47 is an inhibitor of the ATPase activity of p97/VCP, the latter is more likely to remain bound to p47 when ATP is limiting and thus inhibiting hydrolysis [56]. Conversely, binding of p97/VCP to UN promotes retrotranslocation of ER proteins and their ubiquitylation and chaperoning to the 26S proteasome in the ERAD pathway [41]–[43], [57]. This suggests that in an ATP-abundant environment, p97/VCP readily binds to UN over p47, to carry out removal of misfolded proteins or participate in its different roles in the nucleus such as mitotic progression or transcription factor activation. Taken together, competition between p47 and UN for p97/VCP can become heavily biased in certain cells or tissues where the ratio between p97/VCP and these adaptors is altered and ATP is limiting. The physiological relevance of this novel role of ATP in regulating adaptors recruitment and consequent engagement of p97/VCP in either ERAD or homotypic fusion implicates altered metabolic fluxes of ATP as key players. ATP levels may fluctuate within the same cell in different organelles, within different cell types and tissues and may even be affected by age or disease [58], [59]. In this respect, it is interesting that the ability of p97/VCP to bind the Werner syndrome protein is ATP-dependent, suggesting a role for p97/VCP in releasing the Werner syndrome protein from the nucleus [60]. Also in the p97/VCP R155H mutant, a seven-fold reduction in affinity to SVIP was observed when ATPγS was bound. Finally, the importance of the adaptors’ recruitment to the functions of p97/VCP is underscored by the observation that the IBMPFD mutations are located at the hinge region between the N and D1 domains, that the majority of these mutants show higher affinity towards ATP and by the imbalanced binding of adaptor proteins to the IBMPFD mutant of p97/VCP [45]. The importance of ATP levels has been shown in a Drosophila melanogaster IBMPFD model, where a high energy metabolic diet alleviates neurodegeneration [61]. Since p97/VCP is implicated in several diseases including cancer, modulators of p97/VCP can be therapeutic. Our competition assay provides a possible drug screening method, which will allow a high throughput screen for molecules that specifically increase or decrease p97/VCP’s interaction with a particular adaptor protein, and thus achieving a specific and refined form of inhibition than the general pleiotrophic modulation of overall p97/VCP functions.

## Experimental Procedures

### Plasmids and Protein Expression

p97/VCP was cloned into pET-26b (Novagen) *Nde*I and *Xho*I sites generating p97/VCP with a C-terminal His_6_ tag. pQE-p97-N-D1, pET-26b-Ufd1 and pET-30-Npl4 were kindly provided by Professor Hemmo Meyer through the Addgene database (17229,21266,21267,21268). All plasmids were introduced into *E.Coli Rosetta DE3* (Novagen) for IPTG-induced expression of recombinant proteins (0.5 mM IPTG for 4 hours at 0.8A_600_ and 37 °C, with the exception of Npl4 which was induced at 16 °C).

### Protein Purification

Bacteria expressing the respective proteins were pelleted (13,600×*g*, 10 min, 4 °C), resuspended in lysis buffer (50 mM sodium phosphate buffer pH 8/300 mM NaCl/5 mM β-mercaptoethanol including one tablet/50 ml of Complete EDTA-free protease inhibitor (Roche)), sonicated on ice for 10 min (1 sec on/off pulse cycles) cleared by centrifugation (50,000×*g*, 4°C, 20 min) and filtered through a 0.2 µm polyethylsulfone membrane (Nalgene) before loading onto nickel IMAC resin (BioRad; 1 ml of resin per lysate from 1 liter of bacterial culture) for His-Tag affinity purification. The resin with bound proteins was washed with lysis buffer and proteins were eluted with 250 mM imidazole in lysis buffer. Ufd1 and Npl4 were co-purified as a heterodimer through the His_6_-taged Ufd1 that was incubated with an excess of Npl4-containing lysate. Eluted proteins were generally pure enough to be further purified by a single gel filtration step, with the exception of p97-N-D1, which was subjected to anion-exchange to remove contaminants. Before gel filtration, p97-N-D1 was bound to a 1 ml HiTrap Q FF column and eluted by a 0–1 M NaCl gradient in sodium phosphate buffer (pH 8). Gel filtration was carried out on a Superose 6 300/10GL on an Akta FPLC (GE Healthcare) into phosphate-buffered saline (PBS) containing 5 mM β-mercaptoethanol and 0.005% (v/v) p20. The relevant elution peaks were used for SPR binding assays. SDS-PAGE (10%) using Coomassie brilliant blue staining was used to assess protein purity (Figure 1).

### Protein Concentration Measurements

Protein concentration was measured using Bradford assay (Pierce Coomassie Plus Assay kit 23236), and typical regressions of r^2^ = 0.97 to 0.99 for BSA standards curves were used for estimation of samples protein concentrations.

### SPR Binding Assays

Assays were performed at 25°C in PBS containing 5 mM β-mercaptoethanol and 0.005% (v/v) p20 on a Biacore 3000 instrument (GE Healthcare). Proteins to be used as the ligand were immobilized via amine coupling to the carboxy-methyl dextran surface of a Biacore CM5 sensorchip (GE Healthcare). Surfaces were activated for 7 min with a mixture of 0.2 M 1-ethyl-3-(3-dimethylpropyl)-carbodiimide & 0.1 M N-hydroxysuccinimide to convert surface carboxyl groups into an amine reactive ester before the injection of the ligand to be immobilized. Immobilization was performed at pH 4.5 using a flow-rate of 10 μl/min. The control surface was treated in an identical way, omitting the injection of the protein. For binding assays, analytes were injected over the reference and immobilized protein surfaces for 60–120 sec, and allowed to dissociate for 120–140 sec. The flow rate used was 20 μl/min in competition experiments and 30 μl/min for kinetic experiments. ATP, ATPγS, AMP-PNP and ADP were purchased from Sigma-Aldrich (Sigma A26209, A1388, A2647 and A2754, respectively). All nucleotides were prepared as a magnesium-nucleotide complex. Sensorgrams obtained from the experiments were double referenced with the in line reference cell, to subtract bulk effects and non-specific interactions and with buffer injections, and were analyzed using the Biaevaluation 4.1 software (GE Healthcare). Interaction Map analysis (Ridgeview Diagnostics AB, Uppsala, Sweden) of the obtained sensorgrams was performed in TraceDrawer 1.4 (Ridgeview Instruments AB, Uppsala, Sweden) [62]. For competition experiments, p97/VCP captured by p47 immobilized on the sensorchip surface was regenerated/removed between cycles with a 3 sec pulse of 50 mM NaOH, with minimal deterioration of the ligand (p47) for over a hundred cycles. The coinjection experiments were performed using automated functions of the Biacore 3000, namely the automix and coinject functions.

### Dynamic Light Scattering (DLS) Measurements

Dynamic light scattering was performed on a Malvern Zetasizer Nano ZS using non-invasive back scatter (173°C). For monitoring thermal denaturation via DLS, heating was carried out by the built in heating system in the Malvern Zetasizer NanoZS. Poly-methyl-methylacrylate (PMMA) cuvettes were used in all measurements due to thermal stability.

### Differential Scanning Fluorimetry (DSF)

Differential scanning fluorimetry was carried out in a Biorad IQ5 realtime PCR thermocycler, using MicroAmp Optical 8 tube-strips (Applied Biosystems), based on the method of Niesen *et al.* [63]. The results were analysed by Biorad IQ5 software. 2 μM of p97/VCP or p97-N-D1 was mixed in a 1∶1 ratio with 10× Sypro Orange (Sigma Aldrich S5692), dissolved in PBS and the resulting mixtures were incubated on ice for 30 min to allow Sypro Orange to coat the proteins before subjecting 40 μl triplicates to thermal unfolding in the absence or presence of ATP at different concentrations. As a protein unfolds, binding of Sypro Orange to new exposed hydrophobic core regions results in a fluorescent readout monitored by excitation at 492 nm and emission at 610 nm. Protein unfolding in the presence of ligands at various concentrations provides insight into binding and conformational changes resulting in thermostability.

## Supporting Information

Figure S1 **Calibration of Superose 6 10/300 GL column.** The calibration was done using thyroglobulin (667 kDa), ferritin (440 kDa), lactate dehydrogenase (132 kDa) and bovine serum albumin (66 kDa). Elution volumes of the respective proteins (A) and the calibration curve (B) are presented.
(TIF)

Figure S2 **Trial of a 1∶1 Langmuir model fit.** Sensorgrams from the interaction between Ufd1/Npl4 and p97/VCP were poorly fitted to the 1∶1 Langmuir model, suggesting that the interaction may be complex.
(TIF)

Figure S3 **Detailed experimental setup of the competition assay.** The respective sample layout on a Biacore 3000 instrument (A) using the corresponding automix method (B) of the competition assay that permits high reproducibility.
(TIF)

Figure S4 **Dissociation of p97/VCP hexamers by urea.** 1 μM of hexameric p97/VCP was subjected to treatment with the indicated concentrations of urea. Dynamic light scattering measurements reflects dissociation of p97/VCP hexamers starting at 1 M Urea, with increasing intensity of the monomer peak in correlation with the increasing urea concentration.
(TIF)
