# Supplementary figures and images for: ATP Binding to p97/VCP D1 Domain Regulates Selective Recruitment of Adaptors to Its Proximal N-Domain
Source: PLoS One. 2012 Dec 3;7(12):e50490. doi: 10.1371/journal.pone.0050490 (PMC3513293; doi:10.1371/journal.pone.0050490)

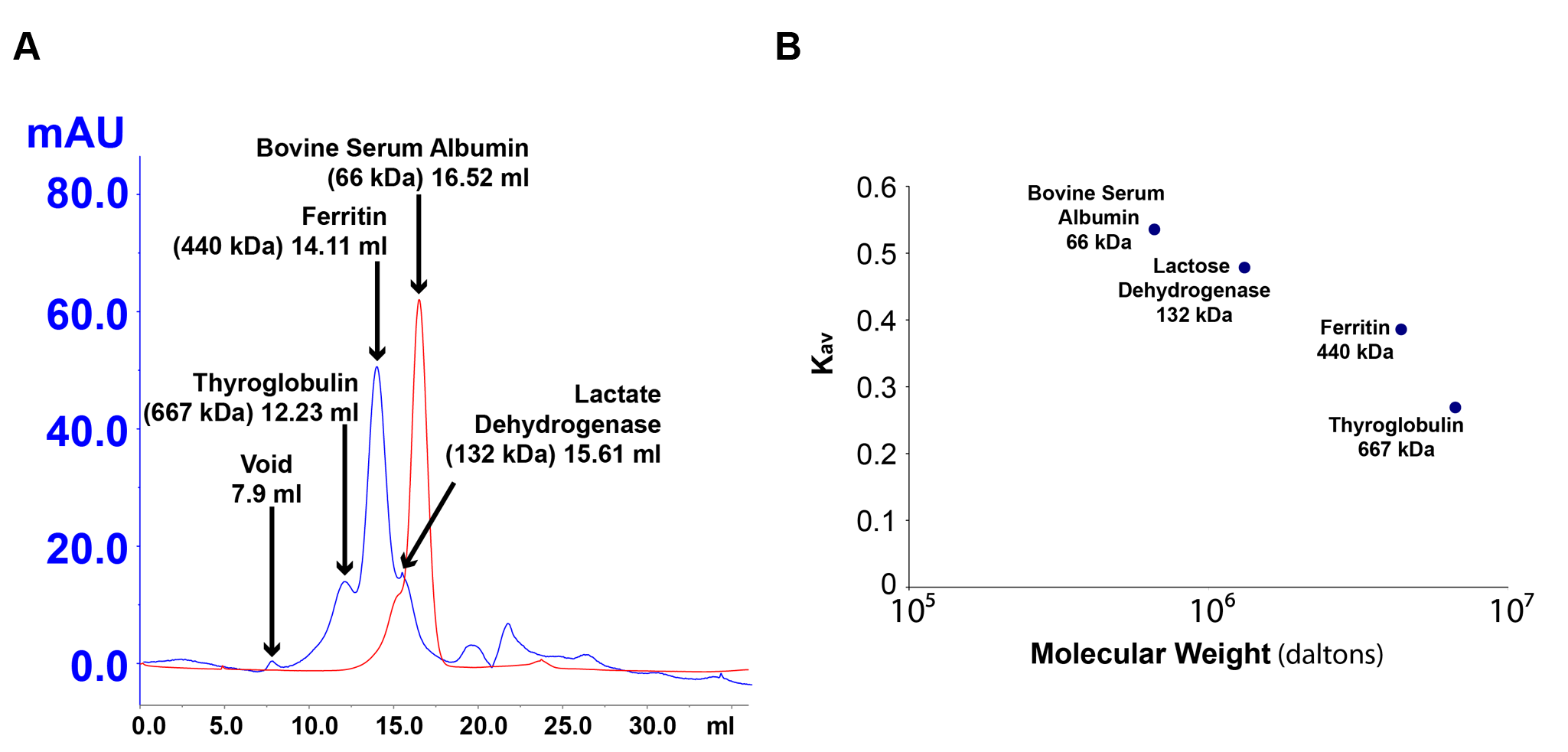

Supplement: Figure S1 — Calibration of Superose 6 10/300 GL column. The calibration was done using thyroglobulin (667 kDa), ferritin (440 kDa), lactate dehydrogenase (132 kDa) and bovine serum albumin (66 kDa). Elution volumes of the respective proteins (A) and the calibration curve (B) are presented. (TIF) [file pone.0050490.s001.tif]

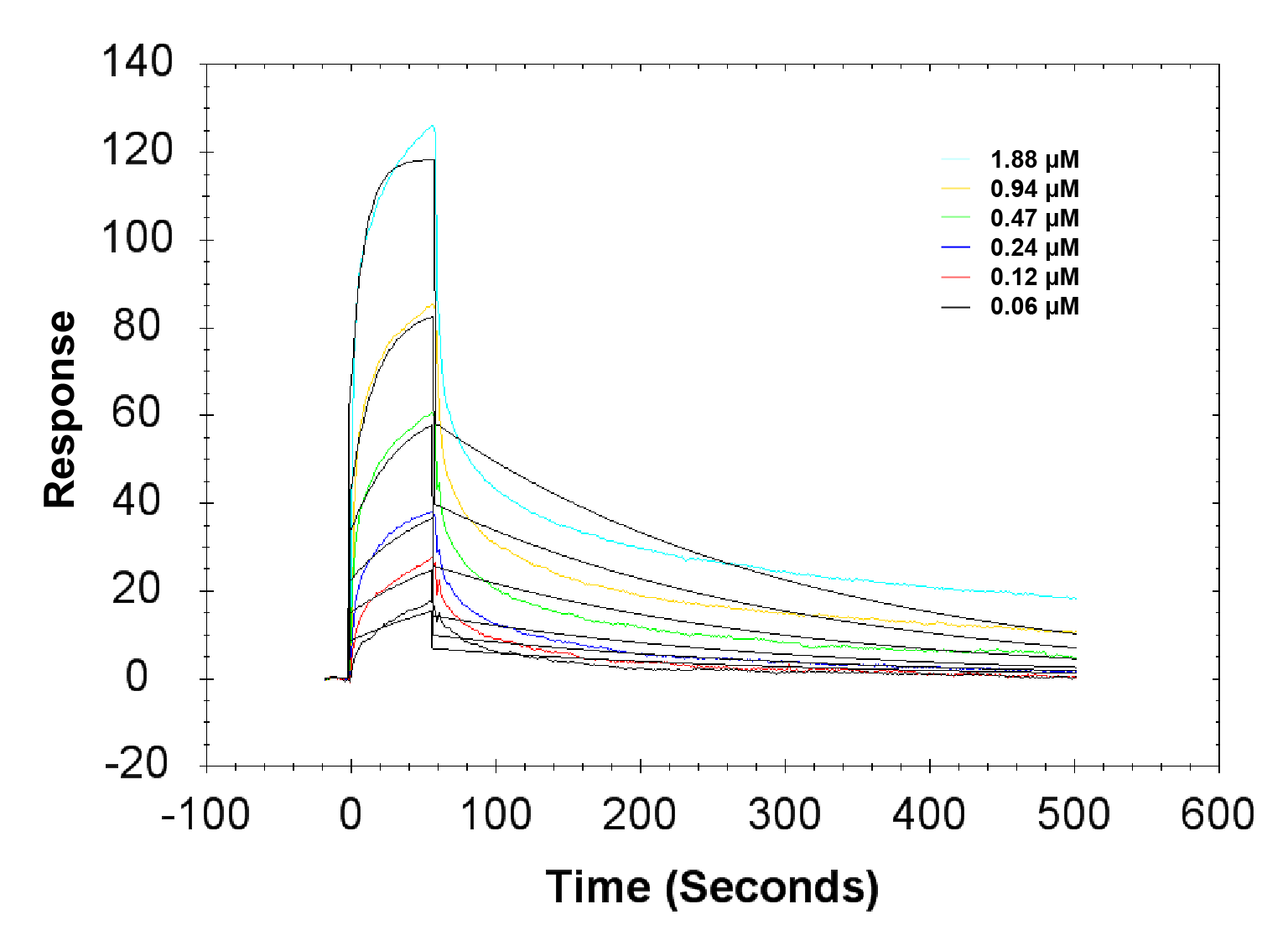

Supplement: Figure S2 — Trial of a 1∶1 Langmuir model fit. Sensorgrams from the interaction between Ufd1/Npl4 and p97/VCP were poorly fitted to the 1∶1 Langmuir model, suggesting that the interaction may be complex. (TIF) [file pone.0050490.s002.tif]

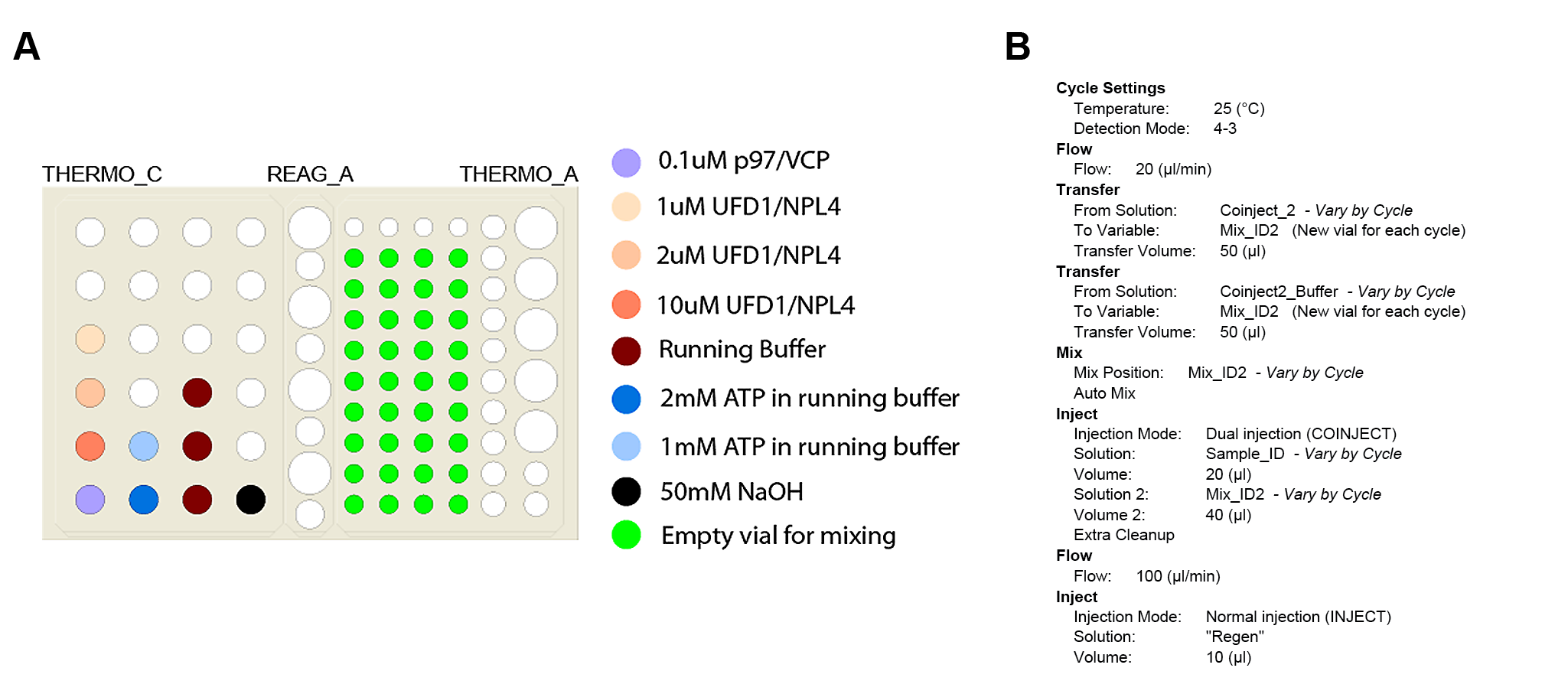

Supplement: Figure S3 — Detailed experimental setup of the competition assay. The respective sample layout on a Biacore 3000 instrument (A) using the corresponding automix method (B) of the competition assay that permits high reproducibility. (TIF) [file pone.0050490.s003.tif]

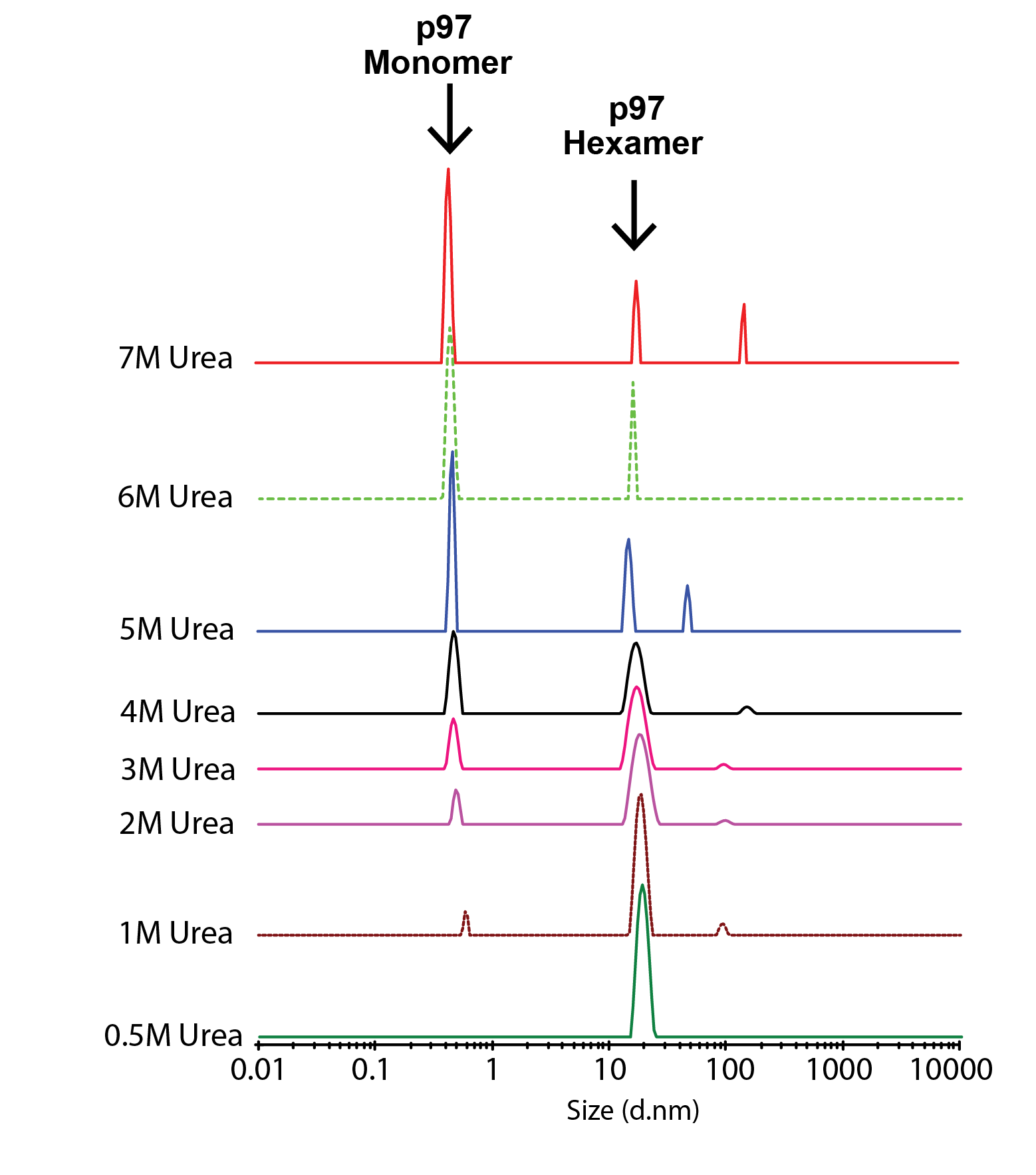

Supplement: Figure S4 — Dissociation of p97/VCP hexamers by urea. 1 μM of hexameric p97/VCP was subjected to treatment with the indicated concentrations of urea. Dynamic light scattering measurements reflects dissociation of p97/VCP hexamers starting at 1 M Urea, with increasing intensity of the monomer peak in correlation with the increasing urea concentration. (TIF) [file pone.0050490.s004.tif]
